# How Elderly Residents in Nursing Homes Handle Loneliness—From the Nurses’ Perspective

**DOI:** 10.1177/2377960820980361

**Published:** 2020-12-17

**Authors:** Prathima Naik, MHSc, Venke Irene Ueland, PhD

**Affiliations:** 1 Faculty of Health Sciences, University of Stavanger, Stavanger, Norway

**Keywords:** loneliness, nursing home, geriatrics, qualitative research

## Abstract

**Introduction:**

Elderly people who leave their home environment and move to a nursing home enter a phase in life with diminishing contact with family and friends. This situation often results in a feeling of loneliness with a concomitant deterioration in physical and mental health. By exploring the topic through the lens of the nurses, this study takes a novel approach to address an under-researched area in the nursing field.

**Objective:**

The objective of the study was to identify, based on the nurses’ experience, how elderly residents handle loneliness in the nursing home.

**Methods:**

This study used a qualitative explorative approach with data collected through two focus group interviews with nine nurses at two elderly care facilities in Norway. The resulting transcripts were examined using an approach based on inductive content analysis.

**Results:**

Three main categories emerged as crucial to help lonely nursing home residents cope with day-to-day life: (i) maintaining ties to one’s earlier life; (ii) engaging in recreational pursuits; and (iii) building new networks.

**Conclusion:**

Analysing the findings based on sense of coherence (SOC) and person-centred care (PCC) theories illustrates the importance of maintaining a connection with both family and friends. To that point, having access to familiar objects from their earlier life seemingly provides meaning to the residents by bridging the past and the present. Recreational activities, ideally adapted to each person’s needs and ability, have a positive impact by providing structure and meaning that help overtake feelings of loneliness. Building a new network with fellow residents and staff imparts a sense of meaningful community belonging and projects both dignity and self-worth.

In 2018, there were 125 million people aged 80 years or older in the world and this population will rise nearly threefold to 434 million by 2050 (WHO, 2018). In Norway, 70% of people older than 80 years live in nursing homes (Drageset et al., 2017). In order to adequately prepare for the millions of people slated to move into nursing homes over the next decades, it is absolutely imperative that society better understands the challenges faced by nursing home residents. Previous research studies have assertively singled out one of these major challenges: loneliness (Paque et al., 2018). When researching elderly’s loneliness, it is of vital importance to seek the nurses’ perspective because they closely interact with the residents and carry a major responsibility for making the residents’ lives better. Weiss identifies two sub-types of loneliness: social and emotional loneliness (Weiss, 1974). Social loneliness refers to an absence (real or perceived) of social networks while emotional loneliness is about the absence of intimate relationships.

## Background

In its most basic form, loneliness is a mental health issue of subjective nature grounded in feelings of distress (Jansson et al., 2017; Theurer et al., 2015). For elderly nursing home residents, loneliness typically refers to a feeling of emptiness in one’s social life caused by death of family or friends, difficulty integrating into a new environment or physical disability impeding interaction with others (Aroh et al., 2016; Drageset et al., 2015; Jansson et al., 2017). However, loneliness in this sense does not necessarily imply an environment devoid of fellow residents – residents can feel lonely even if other residents are physically present in the same place.

In terms of prevalence, loneliness among elderly residents appears to be a relatively widespread phenomenon, at least in the Nordics. Nyqvist et al. suggest that 55% of the elderly residents in care facilities in northern Sweden experience loneliness while Drageset et al. report a corresponding figure of 56% for western Norway (Drageset et al., 2011; Nyqvist et al., 2013). Other international studies estimate that at least one third of older people (to some extent) admit to feeling lonely, but the same studies note that the proportion is higher among people in nursing homes, thus lending support to the figures suggested in the Scandinavian studies (Grenade & Boldy, 2008; Tijhuis et al., 1999).

Social relationships are a fundamental need for a healthy old age, particularly bonds derived from a network, attachment or simply belonging to a group (Eskimez et al., 2019). Although living in a nursing home is meant to reduce loneliness, many elderly still describe a feeling of loneliness with negative implications such as associated loss of social skills and growing social isolation (Morlett Paredes et al., 2019). Ironically, elderly people may feel vulnerable in the nursing home because they reside there to prevent social loneliness but in reality nobody there takes adequate care of them (Eskimez et al., 2019). Loneliness can turn into a vicious circle because a lonely person typically withdraws further from social contexts (Drageset et al., 2015). Previous research has suggested that loneliness leads to several detrimental psychological effects ranging from anxiety to depression (Rönkä et al., 2018). Loneliness has also been identified as a predictor for a deteriorating physical health that may manifest itself as cognitive impairment and higher risk of hospital emergency care (Geller et al., 1999; Jansson et al., 2017). Left untreated, loneliness seems to have serious consequences manifesting itself as a state of general sadness, lack of meaning and lack of motivation, a description supported by several studies that also point to a linkage between loneliness and heightened mortality risk (Drageset et al., 2013; Henriksen et al., 2019; Morlett Paredes et al., 2019).

Social engagements in various forms have been shown to alleviate feelings of loneliness. Practical advice with a positive impact on loneliness include asking the lonely person to teach someone their favourite skill, joining a shared-interest topic group or engaging in animal-assisted therapy programs (Botek, 2020; Brimelow & Wollin, 2017; Gardiner et al., 2018).

How the nurses view loneliness among elderly residents is inexplicably rarely discussed in the literature. It is puzzling that the nurses’ perspective has not been more thoroughly explored considering their role as primary caretakers. These insights offer potential clues as to why the interest from the wider research community in engaging nurses for new knowledge on elderly’s loneliness seems to have been rather muted so far. However, one of the few pertinent research studies on how nurses perceive the experience of lonely nursing home residents revealed difficulties for staff to detect and prevent loneliness in a systematic manner, thus resulting in a living environment with heighted risk that nurses unintentionally overlooked loneliness (Vadseth, 2009).

In summary, the perils of loneliness are a real threat for elderly nursing home residents. Moreover, there is an increasing need to support an ageing population living in institutions in order to improve the overall quality of healthcare services provided to the elderly (Anvik et al., 2020).

The concept of loneliness among elderly has been extensively researched, yet the public domain remains surprisingly thin when it comes to published research incorporating the nurses’ perspective on how to handle loneliness among elderly residents. Suffice it to say, there is a knowledge gap in the research community that arguably requires prompt attention considering the magnitude of existing and prospective lonely residents on a global scale. Hence, the present research study is important and justified in that it aspires to fill this void by mining the knowledge and experience of professional nurses to shed new light on how residents handle loneliness.

### Aims

The aim of this study was to identify, based on the nurses’ experience, how elderly residents handle loneliness in the nursing home. The two key research questions were: (1) How do nurses perceive that elderly nursing home residents handle loneliness? (2) What do nurses do to help the elderly handle loneliness?

### Method

## Design

This study employs a qualitative explorative approach based on two focus group interviews with nurses. The explorative approach is appropriate because little is known about this topic when researched from the nurse’s view. Qualitative methods are often applied to research situations where the objective is to investigate and attain a deeper understanding of individuals’ views and personal experiences (Stewart et al., 2008). These methods are a valuable tool for the project where data collection relies on face-to-face interviews with open-ended questions. The present study collects data through qualitative interviews. As the present study seeks to identify motivational factors reducing, or ideally preventing, loneliness in elderly nursing home residents, it is of principal importance to better understand how the residents feel and perceive their own situation through the lens of the nurses. Both researchers behind this study have a background in social work and nursing, including experience working with elderly in the context of elderly care, mitigating the risk of misconceptions.

## Data Collection

The inclusion criterion required the participants to have at least one year of work experience as a registered (or auxiliary) nurse at a nursing home. We included nurses working only at long-term care facilities whose residents by definition have a longer history of institutionalised care and therefore are more likely to be in a status quo mode. When researching elderly’s loneliness, it is appropriate to access the experience-based knowledge of the nurses because they closely interact with the residents and by doing so gain first-hand insight into their daily life. Furthermore, the preliminary plan included three focus group interviews with five nurses in each to collect as rich a data set as possible given the time and resources available. However, early in the execution phase it transpired that the initial inclusion criteria were not practically feasible in the local region of Norway where the study was to be carried out. The researchers contacted 13 care facilities of which 8 did not reply, 3 declined to participate (citing lack of time and resources) and 2 agreed to support the study by volunteering staff time.

Securing a sufficient number of participants from these two care facilities was possible after assessing that the results of the study would not be materially impacted by such a modification to the plan. Hence, the researchers surmised that recruiting two focus groups with a total of nine participants was adequate. To that point, previous studies on focus group research suggest that over 80% of the data can be derived from having two to three focus groups (Guest et al., 2016, p. 3). When the intra-group dialogue is extensive, two focus groups can provide data sets that are sufficiently rich and relevant to conduct a meaningful analysis (Malterud, 2017; Wibeck, 2012).

The study recruited two focus groups with a total of 9 participants (8 registered nurses, 1 auxiliary nurse) from two nursing home facilities (NH1, NH2). Each focus group consisted of 4 or 5 participants to create a sense of security and help facilitate the conversation, in which all of the participants actively shared their opinions and personal experiences. At NH1, the focus group participants consisted of two male and three female nurses and reported on average more than 10 years of relevant work experience. At NH2, participants consisted of one male and three female nurses (one of whom was an auxiliary nurse) and everyone had more than 10 years’ work experience.

The researcher first contacted the nursing home managers at selected nursing homes via email and enclosed a letter briefly describing the research project and the use of focus group interviews with nurses. The managers replied via email whether their nursing home facility would take part in the study. Once the researcher received confirmation to participate, managers were provided with an electronic copy of the participant consent form and the interview guide. At the same time, the researcher scheduled via email an appointment (time, date, location) for the focus group interview. The managers asked for volunteers before or after committing to participate in the study.

The focus group format chosen was appropriate for the project because it offers the researchers an opportunity to follow up directly with the interviewees to clarify or confirm points or facts that may never have come to light if a mechanical survey template had been used (Leung & Savithiri, 2009). The focus group conversation activated and contributed to developing lines of association and open reflections about the themes (Halkier, 2010; Malterud, 2017). Assisted by a semi-structured interview guide, the primary researcher moderated the focus group discussions which lasted approximately 45 minutes (Malterud, 2017). The interview guide had four questions: 1) *What do you think about loneliness among the elderly at your nursing home*? 2) *How does loneliness manifest itself*? 3) *What do you as a nurse do to help the elderly combat loneliness*? 4) *What do the elderly themselves do to combat loneliness?*

The participants were openly asked about loneliness, without making any further specification of the term. As moderator of the discussion, the researcher tried to facilitate an open conversation and avoided interjecting questions or comments that risked derailing the intra-group talk while at the same time ensuring that the focus remained on the subject. An observer, who was well informed of the study, accompanied the primary researcher to the first focus group interview. The purpose of an observer was to have an independent source assessing how the interview guide worked and whether the information flow dynamics enabled all participants to contribute meaningfully. The observer was in listen-only mode during the focus group interview but provided feedback to the primary researcher after the interview had finished. Note that the observer was not the same person as the second researcher of the study.

The researcher was aware of the shortcomings with the focus group format. For example, the group discussions may be more likely to unconsciously drift toward topics where the participants have a similar view rather than address subjects where participants have widely different opinions to avoid introducing disharmony in the forum (Acocella, 2012, p. 1132). However, the format often inspires more in-depth discussions on specific subjects because each participant can elaborate on what was said previously in the conversation and by doing so adding another layer of insight that may otherwise never have surfaced (Acocella, 2012, p. 1132). Using an interview guide to steer the discussion too rigidly may lead to a loss of content if obedient participants strictly follow the question outline and thus view any adjacent subjects as off-topic (Leung & Savithiri, 2009, p. 218). In our study, the interview guide seems to have been a useful tool for advancing the discussion without curtailing content.

The discussion atmosphere between the researcher and the participants was relaxed. The researcher carefully guided the conversation and helped stimulate dialogue among all the participants without voicing personal thoughts or opinions. No single participant dominated the conversations.

## Data Analysis

The discussions were recorded digitally and later transcribed verbatim. The results were analysed using inductive content analysis. The beauty of qualitative content analysis is that it provides a scientific method to shed light on a social phenomenon that can be both complex and subjective (Zhang & Wildemuth, 2005). The first author was responsible for carrying out the actual data analysis process but both authors discussed, contributed, and jointly reformulated the themes until reaching the final iteration.

First, the focus group transcripts were analysed in a systematic iterative manner based on inductive content analysis where voluminous text is condensed into smaller units that help researchers identify patterns and develop categories to answer the main research question (Elo & Kyngäs, 2008). Thus, the transcripts were read several times to immerse the researcher(s) in the material. Notes of explicit keywords and between-the-lines messages (open coding) were recorded at each iteration. All keywords and phrases were clearly marked using Stemler’s rule of thumb that the word count frequency often provides a good first-degree approximation of relevance (Stemler, 2000). The transcripts were then analysed methodologically in an iterative manner where the researchers broke down chunky pieces of text into finer units (codes) that later combined to form categories that in tandem helped answer the primary research question (Elo & Kyngäs, 2008). At this point, it was imperative to verify that the final main categories indeed reflected the nurses’ experience, i.e., that the components represented the whole (Erlingsson & Brysiewicz, 2017). As recommended by Weber, consistency was the lodestar throughout the coding phase from start to finish in order to ensure that each inference, and in the end the whole study, was designed to support validity and reliability (Weber, 1990).

## Ethical Approval

The study received formal approval (reference number 254912) from the Norwegian Centre for Research Data (NSD). Qualitative research with in-depth penetration of the explored topic calls for special attention to ethical considerations that should permeate every step of the research process. To that point, the researcher took action to protect and preserve anonymity, confidentiality, and the participants' right to withdraw from the study at any time while securing informed consent.

### Results

**Figure 1.**
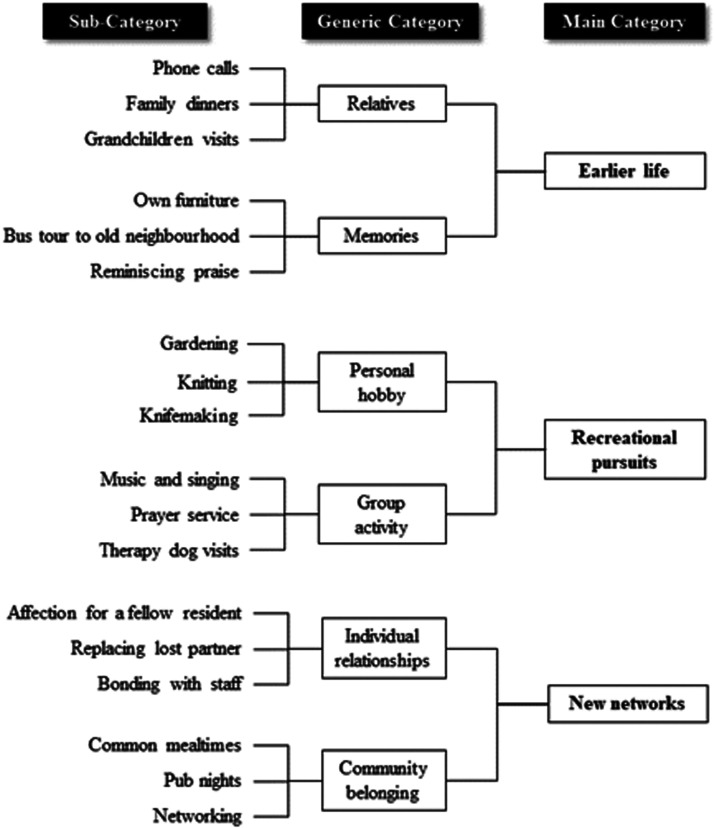
Category Overview of Factors Helping Elderly Residents to Handle Loneliness.

Three main categories emerged as crucial to help lonely residents cope with day-to-day life at the nursing home: earlier life, recreational pursuits and new networks (Figure 1). The findings associated with each of these categories are presented below.

## Ties to One’s Earlier Life

Several participants highlighted the importance of residents maintaining ties with their earlier (pre-nursing home) life as a way to handle loneliness. These ties pertained to both family members and memories of objects that have featured prominently in the previous phase of the residents’ lives.

### Relatives

Maintaining contact with the world outside of the nursing homes stood out as an avenue to handle loneliness. Some residents used the phone as a channel to proactively reach out but also as a conduit to make themselves available for incoming communication. “*The ones with a phone, they sometimes call around, it might be more preventive …*” Several participants called out interacting with family members as a productive way against loneliness. “*… if they have invited the family … and eat with the family, that helps a lot … you see that they’re sparkling when they have been with the grandchildren.”* As long as there was interaction with family, even if it were reduced due to the new circumstances, residents gained mental strength from knowing that the relationships still existed. “*If they are going out, to the family, to their kids, they’re not really lonely … even if the relationship is disrupted it still exists there in the head.”*

There are several ways for residents to maintain ties to their earlier life. They can stay in touch by phone or meet face-to-face with family and friends whom they know from the time before the nursing home. The key to mitigate feelings of loneliness is to somehow relate to the people with whom they have an inner relationship. Just knowing that a connection exists with children, grandchildren or other people helps prevent feelings of loneliness.

### Memories

Several participants emphasised that non-living objects, whether physical or abstract, linked to the earlier life had a positive impact on the residents. There was an opportunity to capitalise on this insight already when a resident moved into a nursing home by furnishing the assigned room with familiar objects to make the resident feel more at home. “*Having furniture, a good chair that they recognise, pictures that they recognise, which show part of their lives that they have lived until now, for many I think that means a lot.”* Another method to preserve memories from the earlier life was for the residents to physically leave the nursing home and visit (by bus) the surroundings of their old home. While this experience was not unequivocally positive for all residents, the participants signalled that the overall satisfaction with this initiative was high. “*That they can drive around where they used to live, it is very nice, prompts memories, even if it can cause anxiety for some, it generates many good things for others.”* Memories of a more abstract nature were also helpful. This sub-category typically involved the nursing home staff talking to the residents about their earlier life to remind them of (the residents’) particular skills or interests, essentially providing a form of reminiscing praise tailored to each patient’s unique background. “… *but those who really express loneliness … to know something about their life story … so you can remind them of whom they have in their lives and what they say … that they brag about you, telling how you always baked delicious cakes.”*

Both physical objects and locations can have a significant impact on reducing the feeling of loneliness. Ties to one’s earlier life are maintained though the connection that exists between physical objects and a person’s life history. Memories are hidden in the connection that links the actual life a person has lived with the mind. Amplifying the connection with one’s past life that is unique to each human being strengthens the individual and can be done by having someone listen to stories about concrete places and things or by keeping memorable physical possessions in the nursing home.

## The Value of Recreational Pursuits

Many of the interviewed nurses underlined the value of participating in recreational pursuits as a means to cope with day-to-day life without descending into loneliness. The emerging pattern showed that these recreational pursuits can be grouped into personal hobbies and group activities.

### Personal Hobby

Enabling the residents to occupy themselves with a pastime pursuit (hobby) of their own liking found significant support among the participants. The actual nature of the hobby was secondary as it was abundantly clear that the participants’ primary focus was on the fact that there was a hobby to begin with, noting that the choice will vary by individual. There was no indication that a solo hobby contributed to a feeling of loneliness. “*Someone is occupied with … cutting out knives, knife blades … and really enjoys doing it. But it is very individual what people like, actually.”* Just being able to do something or create something is in itself a means to reduce loneliness. It provides a way to contribute something concretely and express creativity as a person. This activity provides a sort of breathing space that allows a person to put thoughts about loneliness and the unpleasant situation aside and instead fully dedicate oneself to the pursuit. Personal hobbies reinforce concepts of dignity and self-worth, thereby reducing any feelings of loneliness.

### Group Activity and Network

The elderly taking part in various group activities featured prominently as a response in the focus group interviews. It was evident that the nursing homes in question supported a wide variety of programs in this area as shown in “… *we have very good selection of activities … we have a music café with old music every other week, we have prayer service, we have a reading group …”* and *“singing and music in our section every Friday.”* The popularity of group activities was further demonstrated by the fact that the residents continued with the activity even after the external instructor’s engagement with the nursing home had ended. For example, the residents continued singing together although the music therapy program had officially stopped. The only time the interviews touched on animals was in relation to therapy dogs who were seemingly an appreciated item and part of the formal routine, the so-called activity plan, at both nursing homes. “… *therapy dogs come and some residents really like to pat them and you can see that they are smiling and enjoying themselves.”* Even if nurses perceive loneliness in a resident, they are not necessarily able to take the action needed to get the resident back into a normal mental health state. Apparently, nurses can only motivate – but not force – lonely residents to participate in activities (personal hobbies, family visits, local networking) even if participation would likely reduce the level of loneliness. *“We do have activities, but sometimes they don’t want to participate because not everyone has the same social skills. They sort of keep to themselves for various reasons, so it is not always easy.”* For the nursing staff it is a fine balance between motivating the residents to be active while at the same time respecting their wish for privacy when they prefer solitary to social activities. “*One respects when they say ‘no’, but it is crucial to also motivate and give them opportunities, it’s a balance*.” The dilemma becomes particularly precarious when nurses recognise clear signs of depression, but the resident lacks the willingness to fight back and largely succumbs to the mental slump that may be inescapable without external support. “*The elderly are actually very dependent on the staff…how do they cope when they already are lonely, when they already are depressed? In my view, they can’t cope, the nursing staff have to do something about it, I think…when they are old, they get depressed* *and they start to stay in bed, they don’t want to eat, they don’t want to do anything. How do they cope? I don’t think they can.”* The study participants emphasised the significance of social events. Being together helps the residents to overcome loneliness. A social network with regular gatherings on a variety of topics can break an otherwise monotonous daily life routine. Admittedly, it may be difficult to motivate lonely residents to participate in such activities. Someone may need to kindly nudge them, i.e., give them a friendly push to help them form social connections.

### Individual Relationships

Forming individual relationships with both fellow residents and nursing home staff appeared to be a valuable tool against loneliness. Residents looked to replace a lost partner where lost could refer to either deceased or mentally disabled. This loss created a vacuum that the resident wanted to fill. “*It is something more when you foster a new relationship … especially when one is alone, then one has a need for a close relationship with others. It’s natural.”* Moreover, the participants stated that the formation of new affectionate relationships between fellow residents was more common than many (outsiders) thought while noting that such relationships were more about tenderness and care rather than physical intimacy. “*We also have an example of someone looking for safety or because of loneliness seeks a close relationship with a fellow patient.”* A feeling of (male) responsibility, akin to a breadwinner’s mentality, was another contributing factor to the formation of relationships among fellow residents. “*You have males who have been the strong person in the relationship all their lives, then you lose, a loss, that you try to cover, and you end up in the relationship that just shows your sense of responsibility.”*

The participants answered in harmony that talking to the residents and perusing their background documentations were effective means to build relationships as a measure against loneliness. “*Talk to them. Try, for instance, if someone stays in their room, walk in to them … ask how things are going … try to ask a few questions, read up on the patient’s situation, maybe some background information.”* It emerged that the dependents are also considered essential in forming new relationships with the residents. “*Get to know each other, well, get to know the dependents. The dependents are equally important to get to know the resident who is here.”* Finally, non-verbal cues were important for getting to know the residents and building up a sense of safety and companionship. “*It can be the look, doesn’t blindly have to be the language … it can be the appearance, it can be how they walk, how they sit, how they eat …”*

It is important that the elderly form social bonds also with specific individuals. Knowing some people better than others provides a special dimension of what it means to be a unique human being and mean something to others. It may be a way to relieve feelings of loneliness. Nurses and other health care professionals who share some personal stories with the elderly can also have a positive impact that helps mitigate loneliness.

### Community Belonging

The sense of belonging to a group, being part of a community, permeated the discussions in several ways. First, some participants pointed out that simply living day-to-day in an environment surrounded by other people brought a sense of belonging to a community compared to spending time alone at home. “*And at the nursing home there are at least the staff and there are other fellow residents … I sometimes think that the loneliness is about affiliation. To feel affiliated with the ones you have around you …”* In fact, elderly on short-term stay in the nursing home sometimes appreciated the community and the camaraderie to such an extent that they would rather have stayed there than returned home. “*We see it especially on short-term stay because they are admitted and then they don’t want to go home again because it’s lonely at home, they thrive when they are together with the rest of us.”*

Second, participants described the common meals as particularly valuable from a loneliness perspective to strengthen the bonds between the members of the community. The meals constituted a natural avenue toward building the community in that they followed a regular schedule, provided a meeting place for staff and residents and resembled the long-held tradition among many of today’s elderly that the family typically gathered at mealtime. “*Recently, we have a community lunch for the whole house once a week … For everyone. It includes the staff because it is an important factor that the staff can sit and eat together with the patients, it gives more of a sense of community.”* In the same vein, summer barbeques and pub nights were other meal-related activities of a collective nature that enjoyed widespread support.

Third, seeking contact – networking – within the community was a way to avoid loneliness. Some residents actively looked for more involvement in the community as if they were longing for a sense of belonging. However, it was not a universal attribute because some residents were impaired by their health and others by nature had a more introvert personality. “*Those who have language and mobility, they, many look for a community by themselves, but it varies quite a bit because it’s a very personal issue.”*

Residing in a nursing home in itself creates a sense of belonging. To be a part of the nursing home environment can make one feel safe, cared for and surrounded by familiar faces. It also provides routines and a system. That in itself can give rise to less loneliness than in a home environment where one is left alone, perhaps without having any close connections around. The structure, design and form of the actual institution also contribute to residents feeling more at home.

## Discussion

The aim of this study was to draw on the nurses’ experience to illuminate how elderly residents handle loneliness in the nursing home. The current findings identified three main areas helping the elderly residents handle loneliness: maintaining ties to one’s earlier life, engaging in recreational pursuits and building new networks. These results lend themselves particularly well for a deeper analysis using the sense of coherence (SOC) and person centred care (PCC) theories because these theoretical frameworks encapsulate the focus on individuals coupled with their ability to handle an adverse experience (loneliness). In PCC, the goal is to meet each unique individual’s subjective needs by focusing on the person’s resources, conditions and limitations (Kitson et al., 2013). Antonovsky’s theory of SOC concentrates on subjective well-being in spite of illness and losses in life while considering an individual’s health resources (Antonovsky, 1987).

The first observation from the present study was that familiar people and personable belongings associated with the resident’s earlier life play a vital role in the handling of loneliness because they presumably provide a bridge between the past and the present. Upholding interpersonal relationships with family and friends has previously been shown to play an important role for residents’ well-being and arguably represents the SOC component of ‘meaningfulness’ (Malini et al., 2018; Rice, 2012). Viewed through the prism of SOC it seems plausible that maintaining such bonds contributes to making the resident’s life at the nursing home (more) meaningful and by extension less lonely.

The study appears to show that finding links to memories in the past imparts meaning to one’s present life. This bridge between the past and present lives may reduce social loneliness. Even if the ties to the people in past life episodes do not necessarily represent close relationships, they may still suffice to fill a basic need in this context. In our view, just being aware of the people who have had an influence on one’s life can create the support needed to cope with the situation. Maintaining social ties through memories of people and places of significance from the past is important to stave off loneliness. Indeed, Weiss claims that social loneliness rather than emotional loneliness leads to depression among elderly people (Weiss, 1974). Loss of social skills in the present life increases the need to preserve memories of one’s social life in the past (Morlett Paredes et al., 2019).

The nurses’ reference to personal objects in the rooms evoking memories of the past weaves together both SOC and PCC. Doyle and Roberts contend that such objects transmit a special meaning (as in ‘meaningfulness’) while Fazio et al. stress the role of personal possessions in individualised care (Doyle & Roberts, 2017; Fazio et al., 2018). Vikström et al. exemplify PCC theory in practice by citing national person-centred care guidelines that recommend decorating the rooms with memorabilia (Vikström et al., 2015).

Given the importance of PCC and maintaining pre-nursing home relationships with family and friends as suggested by SOC, it is striking that the use of modern communication technology such as the Internet, smart phones and video conferences still seems to be virtually non-existent among the residents. Similarly, nurses highlighted the value of putting familiar pictures in the residents’ rooms but there was no mention of tablets for automatically displaying hundreds, if not thousands, of photos from the past. These findings corroborate the conclusion from previous studies which found that digital communication channels were under-utilised (Vadseth, 2009; Mutafungwa, 2009).

Second, the study identified recreational pursuits as a key motivational factor helping residents handle loneliness. Scott argues that daily activities are valuable because they instil a pattern of stability and predictability (Scott, 2009). Building on Scott’s argument, it seems likely that the cited so-called activity schedule at the nursing homes could have an outsized positive impact on loneliness by providing structure and predictability to the residents’ daily routine, reflecting the element of ‘comprehensibility’ in SOC theory (Rice, 2012). Interestingly, an academic study has shown that routine visits by family and others to the elderly residing in nursing homes reportedly strengthen the elderly people’s self-respect (Drageset et al., 2015). Another logical interpretation of this finding that mirrors the reasoning of SOC’s element of ‘meaningfulness’ is that residents who are busy with an activity they genuinely enjoy are present in the moment and have less time to dwell on the past and feel sad, i.e., the recreational pursuits create a meaningful environment (Mohammadinia et al., 2017).

Loneliness in residents’ social life typically refers to a feeling of emptiness caused by losing those near and dear or living in an unfamiliar environment (Aroh et al., 2016; Drageset et al., 2015 ). The findings of the study show that the feeling of emptiness can be reduced through various regular activities and social gatherings that create a pattern of stability and predictability in the residents’ daily routine.

These findings, at least in part, mirror those of other studies that report some success in reducing social isolation and loneliness among older people through participation in various activities. This type of engagement seems to increase social connectedness (Botek, 2020; Brimelow & Wollin, 2017; Gardiner et al., 2018).

A crucial underlying assumption in this reasoning is that the residents enjoy the activity. But as suggested by the participants, and corroborated by other research, having a large age gap of up to a generation as well as varying individual preferences means that scheduling common group activities suitable for all residents would not be an effective measure to form a meaningful everyday life (Holst et al., 2018). In a similar vein, participants echoed insights from Laffon de Mazières et al. that the degree of disability seems to be increasing over time for incoming residents, thus progressively narrowing the types of hobbies and activities appropriate for most residents (Laffon de Mazières et al., 2017). In practice, the activities should therefore as prescribed by PCC be tailored to each resident’s needs and preferences to the extent practically and economically feasible. As an additional benefit of more person-centred activities, residents would likely sense a greater feeling of support and control as stipulated by the element of ‘manageability’ in SOC (Rice, 2012). This double benefit could create a positive feedback loop, a virtuous circle, amplifying the positive effect on supressing any feelings of loneliness.

Third, the study identified building new networks as an essential mechanism to handle loneliness. One dimension of this mechanism is creating a sense of belonging to a community. Referring to Weiss, social loneliness relates to loneliness caused by dissatisfaction with one’s social network or a more general social isolation. This means that having a social network is a precondition for remediating emotional loneliness (Weiss, 1974). This finding exemplifies what Baumeister and Leary describe as a fundamental need for humans to “belong to a social group” – a need on par with other basic needs such as sleep or hunger to avoid loneliness (Forsyth, 2014). Such social support network has indeed been shown to directly reduce loneliness among elderly in nursing homes (Winningham & Pike, 2007).

In our view, these networks create structures that can fill a void in the residents’ everyday life and may induce a feeling of living in the present that suppresses loneliness. Admittedly, this view is not unchallenged. While our study shows that being around other people in a nursing home helps combat isolation, other studies contend that one can still feel lonely and isolated even in the company of others (Morlett Paredes et al., 2019).

Establishing new networks also underpins the applicability of SOC theory as these provide vital support and resources along the element of ‘manageability’. Koelen’s research takes it one step further by suggesting that belonging to a community touches on all three elements of SOC with the component of ‘meaningfulness’ derived from the insight that networks generally enhance older people’s perceptions of having a purpose in life (Koelen et al., 2017). While nurses generally praised the common mealtimes as an avenue to build a sense of community belonging, they also respected the wish of some residents to eat by themselves in their rooms. Acknowledging such individual preferences aligns with the tenets of PCC theory.

Another dimension worth highlighting is the depth of the new networks. Simply living in a nursing home surrounded by other people is not sufficient to ensure inclusion in social networks preventing loneliness (O’Rourke et al., 2018). Indeed, this study suggested that the critical factor against loneliness primarily lies in establishing and sensing belongingness to new networks. The contention here is that just sensing the belongingness reflects SOC’s ‘meaningfulness’, making the actual depth of the network secondary. To illustrate this concept in the present study, residents formed new relationships with fellow residents of the opposite gender to have someone to care for and to hold hands with, but not for deeper physical involvement. In the same way, receiving attention and getting to know the staff projects self-worth, dignity and respect for the residents. Even short conversations appear to be a powerful tool in reducing loneliness, possibly signalling the value of PCC. Conversely, as a result of inverting SOC’s ‘meaningfulness’ and reducing the PCC impact, the lack of a network should logically have the opposite effect, i.e., it should amplify loneliness. This prediction actually matches previous studies which found a link between loneliness and residents feeling dissatisfaction, being uncared for or having limited interactions with staff (Lung & Liu, 2016; Slettebø, 2008).

### Methodological Considerations and Study Limitations

Qualitative study findings are not necessarily (wholly) transferable; admittedly, the regional concentration of the two Norwegian nursing care facilities in the data sample represents one such limiting factor (Malterud, 2001). It is also conceivable that a larger number of participants may have yielded an even richer data set. However, as Malterud points out, bigger data sets do not necessarily translate into more accurate scientific insight as long as the overall study is judiciously designed (Malterud, 2001). As recommended by Weber, consistency was the lodestar throughout the coding phase from start to finish in order to ensure that each inference, and in the end the whole study, was designed to support validity and reliability (Weber, 1990).

Reliability and validity in the context of non-positivist research is understood as dependability and credibility, respectively. The dependability is demonstrated through crisp and clear presentation of the findings as well as of the steps in the content analysis (see Figure 1), both of which contribute to making the analysis more transparent and thus increasing the rigor of the study. The credibility of the study is strengthened by the fact that both authors participated and discussed the preliminary thematised data to refine the interpretation and reach a consensus regarding the information gathered. Further comparing and contrasting the findings of the study to those of relevant previous research studies also increases the credibility. We suggest that our findings can support the understanding of how elderly residents in nursing homes handle their loneliness.

The thorough analysis thus conforms to the purpose and benefits of content analysis; that is, it conveys the participants’ true underlying message without being tainted by undue pre-understandings or constrained theoretical frameworks (Hsieh & Shannon, 2005).

The discussion of the findings in light of sense of coherence (SOC) and person centred care (PCC) theories validated the three identified main categories and has proven to be a suitable framework for deriving some universal knowledge on the topic (Malterud, 2017). However, we need to develop more knowledge about how healthcare professionals dealing with the elderly perceive and experience the residents’ feelings of loneliness.

## Conclusion and Implications

Drawing on the nurses’ experience in a focus group format and evaluating the findings in light of the SOC and PCC theories, this qualitative research study demonstrated that three factors spanning multiple dimensions interact to help elderly nursing home residents handle loneliness: earlier life, recreational pursuits and new networks.

The research contribution to existing knowledge is a complementary understanding of elderly’s loneliness since it is viewed through the lens of the nurses. The nurses in the study seem to be aware of the residents’ need to bridge their past and present lives as a way to reduce loneliness. The study adds value by extending knowledge about how the gap between past and present can be bridged, at least to some extent, by maintaining links to memories in the past, physical objects and familiar locations. Maintaining a connection with both family and friends while having access to familiar objects from their earlier life provides meaning to residents by bridging the past and the present. Recreational activities, ideally adapted to each person’s needs and ability, have a positive impact by providing structure and meaning that help overtake feelings of loneliness. Building a new network with fellow residents and staff imparts a sense of meaningful community belonging and projects both dignity and self-worth. From a practical standpoint, measures to mitigate loneliness likely require an integrated approach addressing all of these three categories.

Future studies should seek to replicate this study but involve actual residents rather than nurses as the primary information source in order to verify the validity of the findings in this paper and further advance the knowledge of the field. Additionally, exploring how digitalisation can best help reduce loneliness among nursing home residents seems to merit further research.
